# Supplementary material for: KG2ML: integrating knowledge graphs and positive unlabeled learning for identifying disease-associated genes
Source: Front Bioinform. 2026 Jan 8;5:1727953. doi: 10.3389/fbinf.2025.1727953 (PMC12823822; doi:10.3389/fbinf.2025.1727953)
Supplement: Supplementary file 1 [file Supplementaryfile1.pdf]

KG2ML: Integrating Knowledge Graphs and Positive Unlabeled Learning for Identifying Disease-Associated Genes

Praveen Kumar[1], Vincent T. Metzger[1], Swastika T. Purushotham[1], Priyansh Kedia[1], Cristian G. Bologa[1], Christophe G. Lambert[1], Jeremy J. Yang[1]
[1]University of New Mexico (UNM), School of Medicine, Department of Internal Medicine, Translational Informatics Division, Albuquerque, New Mexico, USA

Supporting Information

Table SI-1 presents the top 15 genes with the highest calibrated probabilities of association for each of the 12 diseases analyzed in this study. Validation through a comprehensive review of existing scientific literature and the TINX database confirmed that many of these top-ranked genes are indeed associated with their respective diseases. This result highlights the effectiveness of PU learning in identifying potential disease-gene associations that are not explicitly represented in the knowledge graph.

Gene	Calibrated probability	Associated with bipolar disorder?
XPC	0.8870	No
ZBTB16	0.8754	Yes
SPOP	0.8734	No
STAT6	0.8730	Yes
MRE11	0.8705	Yes
GJC2	0.8697	Yes
LIMK1	0.8695	Yes
RFC2	0.8677	No
MLXIPL	0.8673	Yes
LAMC2	0.8655	Yes
PKP2	0.8647	Yes
PRKCD	0.8602	Yes
NCF1	0.8592	Yes
DDR2	0.8588	Yes
PICALM	0.8571	Yes

Gene	Calibrated probability	Associated with coronary artery?
CCND1	0.9195	Yes
DDB2	0.9040	Yes
HABP2	0.9003	Yes
FGFR3	0.8917	Yes
SLC2A3	0.8913	Yes
MAP3K8	0.8861	Yes
RAD54B	0.8832	No
BBS1	0.8829	Yes
KMT2D	0.8779	Yes
JAK2	0.8772	Yes
FASLG	0.8768	Yes
PROC	0.8761	Yes
TBL2	0.8684	Yes
TERT	0.8680	Yes
EIF2AK3	0.8678	Yes

Gene	Calibrated probability	Associated with diabetes?
PRKN	0.8967	Yes

Gene	Calibrated probability	Associated with diabetic neuropathy?
DLC1	0.5709	Yes

FASLG	0.8896	Yes
RAD54B	0.8877	No
CCND1	0.8869	Yes
KRAS	0.8798	Yes
RAF1	0.8764	No
IFNG	0.8762	Yes
LAMC2	0.8735	No
BCL10	0.8727	Yes
TERT	0.8714	Yes
SPOP	0.8660	Yes
KMT2D	0.8654	Yes
STAT4	0.8646	Yes
CC2D2A	0.8609	No
MRE11	0.8600	Yes

PKP2	0.5413	No
LRRK2	0.5407	Yes
SDHAF2	0.5401	No
TMEM127	0.5366	No
SLC25A11	0.5356	Yes
BAP1	0.5354	Yes
KIF1B	0.5331	Yes
NR2F2	0.5298	No
KEAP1	0.5298	Yes
CUX1	0.5280	Yes
GATA4	0.5275	No
KMT2D	0.5260	No
FANCE	0.5258	No
SLC22A18	0.5255	No

Gene	Calibrated probability	Associated with hodgkin lymphoma?
SLC2A3	0.8704	Yes
PKHD1	0.8700	No
FASLG	0.8699	No
VEGFC	0.8693	Yes
PEX11B	0.8664	No
LRP5	0.8610	Yes
BMI1	0.8609	No
IARS2	0.8554	No
XPA	0.8520	Yes
NTRK1	0.8458	Yes
NUP214	0.8451	Yes
RASGRP1	0.8451	Yes
PTPRJ	0.8448	No
GJC2	0.8435	No
DCX	0.8415	No

Gene	Calibrated probability	Associated with hyperaldosteronism?
LAMB3	0.8770	No
DDR2	0.8677	No
HNF1A	0.8548	Yes
HSD11B2	0.8543	Yes
NFKB2	0.8531	Yes
PRKCD	0.8442	No
BMI1	0.8431	Yes
CYP21A2	0.8311	Yes
GPC4	0.8310	Yes
DOCK8	0.8308	No
GPR35	0.8270	No
SEC31A	0.8269	No
RAF1	0.8238	Yes
FGFR3	0.8234	Yes
ARL6	0.8218	No

Gene	Calibrated probability	Associated with kidney failure?
ATM	0.9122	Yes
FGFR3	0.9066	Yes
KEAP1	0.9007	Yes
PIK3R1	0.8965	No
FASLG	0.8964	Yes
CCBE1	0.8909	No
IKBKG	0.8907	No
FBN1	0.8871	Yes

Gene	Calibrated probability	Associated with malaria?
XPA	0.9457	Yes
NTRK1	0.9451	No
HTT	0.9440	Yes
NFKB1	0.9429	Yes
DDB2	0.9382	No
CACNA1C	0.9381	No
PLA2G2A	0.9326	Yes
TFE3	0.9310	No

ADAMTS2	0.8833	No
LRRC8A	0.8829	No
AKT1	0.8802	No
RAD51C	0.8789	No
ZBTB16	0.8775	No
RB1	0.8754	No
PRKN	0.8748	No

BCL10	0.9304	No
RAD51C	0.9257	No
TBL2	0.9227	No
F10	0.9202	Yes
TTC37	0.9181	No
LIMK1	0.9154	No
FASLG	0.9129	No

**Gene**	**Calibrated probability**	**Associated with Parkinson's Disease?**
TP63	0.9373	No
APC2	0.9333	No
DDB2	0.9315	No
SLC22A18	0.9297	No
FASLG	0.9265	Yes
F2	0.9256	No
CASP8	0.9241	Yes
TMEM237	0.9234	No
BUB1B	0.9227	Yes
NTRK1	0.9172	Yes
ERCC4	0.9159	No
SOX5	0.9159	No
FOXP3	0.9159	Yes
TRPV3	0.9153	Yes
GJC2	0.9148	Yes

**Gene**	**Calibrated probability**	**Associated with pulmonary edema?**
RAD54B	0.8654	Yes
COX7B	0.8613	Yes
LAMB3	0.8595	No
NOTCH3	0.8486	Yes
KCNT1	0.8443	No
ERCC3	0.8428	No
AXIN2	0.8374	No
FERMT1	0.8351	No
XPA	0.8338	Yes
FGFR3	0.8335	Yes
SCNN1G	0.8275	Yes
SLC18A3	0.8257	No
NR2F2	0.8218	Yes
KEAP1	0.8214	Yes
CCND1	0.8168	Yes

**Gene**	**Calibrated probability**	**Associated with raynaud's syndrome?**
SRC	0.7545	No
PLA2G2A	0.7495	Yes
GP1BB	0.7301	No
ASCL1	0.7298	No
ZNF408	0.7291	No
UFD1	0.7227	No
SEC24C	0.7203	No
GPR35	0.7197	Yes
GNPTAB	0.7168	No
OGG1	0.7114	Yes
JMJD1C	0.7101	No
TBX1	0.7087	No
ZFP57	0.7073	No
CISH	0.7060	No
CUX1	0.7050	No

**Gene**	**Calibrated probability**	**Associated with Schizophrenia?**
PRKG1	0.6469	Yes
SLC6A19	0.6446	Yes
GJC2	0.6364	Yes
DOCK8	0.6251	Yes
SETD2	0.6226	Yes
FOXE3	0.6207	No
BCL10	0.6202	Yes
ANAPC1	0.6155	Yes
HNF1A	0.6126	Yes
TRPV3	0.6042	Yes
ERBB2	0.6039	Yes
SCNN1G	0.6037	Yes
RFC2	0.5921	Yes
PTPN12	0.5919	Yes
SOX18	0.5909	Yes

Table SI-1: Top 15 genes with the highest calibrated probability of association for each of 12 diseases, as estimated by the PULSCAR method. Notably, several of these genes are associated with their respective diseases, even in the absence of explicit links in the knowledge graph.

Figure 2 Cypher Queries:

Chronic heart failure in DDKG (Panel A):

```
MATCH (d:Concept )-[dc: CODE]-(d1:Code
{SAB:'SNOMEDCT_US'})-[pt:PT]-(e:Term)
MATCH (c:Concept )-[cc: CODE]-(c1:Code {SAB:'PUBCHEM'})
MATCH (g:Concept )-[gc: CODE]->(g1:Code{SAB:'HGNC'})
MATCH (d)-[r:indication {SAB:'IDGD'}]-(c)-[r1]-(g)
WHERE e.name="Chronic heart failure"
RETURN d as diseaseConcept, dc as diseaseCode, d1 as snomedCode, pt
as diseaseTerm, e as diseaseName,
c as compoundConcent, cc as conceptCode, c1 as pubchemCode, g as
geneConcept, gc as geneCode, g1 as hgncCode, r as relIndication, r1
as activityName
UNION
MATCH (d:Concept )-[dc: CODE]-(d1:Code
{SAB:'SNOMEDCT_US'})-[pt:PT]-(e:Term)
MATCH (c:Concept )-[cc: CODE]-(c1:Code {SAB:'PUBCHEM'})
MATCH (p:Concept )-[pc: CODE]-(p1:Code {SAB:'UNIPROTKB'})
MATCH (d)-[r:indication {SAB:'IDGD'}]-(c)-[r1:bioactivity]-(p)
WHERE e.name="Chronic heart failure"
RETURN d as diseaseConcept, dc as diseaseCode, d1 as snomedCode, pt
as diseaseTerm, e as diseaseName,
c as compoundConcent, cc as conceptCode, c1 as pubchemCode, p as
geneConcept, pc as geneCode, p1 as hgncCode, r as relIndication, r1
as activityName
```

Nodes: 1,077
Relationships: 13,150

Chronic heart failure in Condensed-KG (Panel B):

```
MATCH (d:Disease )-[r:indication ]-(c:Compound )-[r2]-(a:Gene)
```

```
WHERE d.node_label = "Chronic heart failure"
RETURN d as DiseaseName, r as relIndication, c as CompoundName, r2 as
activityName, a as GeneName
UNION
MATCH (d:Disease )-[r:indication ]-(c:Compound
)-[r1:bioactivity]-(b:Protein)
WHERE d.node_label = "Chronic heart failure"
RETURN d as DiseaseName, r as relIndication, c as CompoundName, r1 as
activityName, b as GeneName
```

Nodes: **214**
Relationships: **460**
